# Reassessing ^177^Lu-PSMA-617 Acquisition Costs: Implications for U.S. Radiopharmaceutical Therapy Cost-Effectiveness Analyses

**DOI:** 10.2967/jnumed.126.272999

**Published:** 2026-11

**Authors:** Kyle Flannery, Paul F. Cavanaugh, Kevin Steele

**TO THE EDITOR:** We read with interest the recent publication by Gogebakan et al. (1) evaluating the cost-effectiveness of ^177^Lu-PSMA-617 versus cabazitaxel from a U.S. health care perspective.

We commend the authors for addressing a relevant health economic question. We agree with their conclusion that ^177^Lu-PSMA-617 is unlikely to be cost-effective versus cabazitaxel under conventional U.S. willingness-to-pay thresholds. However, one key modeling input, ^177^Lu-PSMA-617 acquisition cost, merits further consideration because it may understate the economic difference between these therapies.

The authors note that direct U.S. cost data for ^177^Lu-PSMA-617 were not available and therefore used the price of ^177^Lu-DOTATATE as a proxy, citing a pan-Canadian Oncology Drug Review (pCODR) report. It is important to clarify that the cited pCODR report did not generate independent cost estimates but rather reviewed an economic model submitted by the pharmaceutical manufacturer, Advanced Accelerator Applications (2). Thus, the ^177^Lu-DOTATATE cost inputs are derived from manufacturer-submitted pricing assumptions rather than de novo pCODR analyses. This adds uncertainty because the model relies on a different product, manufacturer-derived pricing, and a non-U.S. health system. Because Canadian drug prices are systematically lower than U.S. prices, this proxy would be expected to bias acquisition costs downward.

The modeled cost assumptions also appear conservative relative to the broader range acknowledged in the article (1). The base-case per-cycle cost for ^177^Lu-PSMA-617 was $32,062, with a sensitivity range of $25,649–$38,474, reflecting a 20% variation around the base case (i.e., 80%–120%), consistent with the underlying Advanced Accelerator Applications model. The pCODR report explicitly critiqued this method, noting that “many of the variables included in the probabilistic sensitivity analysis are varied by an arbitrary factor, such as 20% … good modeling practice would dictate the use of observed values rather than assumed variation” (2). The authors also cite published estimates suggesting U.S. per-cycle costs may range from $25,000 to $75,000, depending on the site of care and provider (1). Accordingly, the upper bound of the sensitivity analysis ($38,474) is approximately 49% lower than the $75,000 upper bound cited in the article (1) and 31% lower than the base estimate of $55,522 per dose derived from U.S. reimbursement data in our recent analysis (3). Because acquisition cost is a principal driver of cost-effectiveness, a narrow, assumption-driven range may compress model results. Although the directional conclusion is unlikely to change, the magnitude of difference is likely greater than reported.

We also question the statement that the ^177^Lu-DOTATATE proxy serves as a transparent interim input “until the reimbursement cost for ^177^Lu-PSMA-617 in the United States becomes available.” This framing does not fully reflect U.S. radiopharmaceutical reimbursement. Unlike many Medicare Part B drugs, radiopharmaceuticals do not follow a uniform average sales price (ASP)–based pathway. Although some therapeutic radiopharmaceuticals in the hospital outpatient setting may be reimbursed under ASP-based methods, guidance from the Centers for Medicare and Medicaid Services states that radiopharmaceuticals furnished outside the hospital outpatient department are not subject to ASP. In those settings, Medicare Administrative Contractors (MACs) determine payment limits using methodologies in place on or before November 2003, including invoice-based or locally derived pricing approaches (4–6). Thus, reimbursement may vary by site of care and MAC jurisdiction rather than reliance on a centralized ASP benchmark. Although ^177^Lu-PSMA-617 was approved by the Food and Drug Administration in March 2022 (7), there remains no ASP-based benchmark for this agent within the CMS ASP framework; under the current reimbursement structure, such a benchmark would not be expected to emerge absent U.S. legislative or regulatory change (4–6).

Given this structure, alternative proxy methods may better reflect real-world U.S. payment levels than cross-product or international pricing assumptions. In a recent analysis presented at the 2026 American Society of Clinical Oncology Genitourinary Cancers Symposium (3), we estimated the U.S. cost of ^177^Lu-PSMA-617 using publicly available MAC reimbursement data. We collected all identifiable MAC payment rates for ^177^Lu-PSMA-617 (HCPCS A9607) across publicly available MAC jurisdictions (JE, JF, JJ, JM, J6, JK, and J15), standardized rates to a per-millicurie basis, and used the median to reduce outlier influence. Assuming the use of a 200-mCi dose, this yielded an estimated per-dose cost of $55,522 (3). This method uses observed U.S. reimbursement and avoids cross-product or cross-country proxies.

These considerations highlight a persistent challenge in U.S. radiopharmaceutical economic evaluations: limited price transparency. Published cost estimates for ^177^Lu-PSMA-617 vary widely, and despite our targeted searches, we are not aware of a clearly reported, centralized manufacturer-provided estimate of the average cost in the United States. In this context, U.S.-specific methodologies are particularly important.

We commend the authors for an important contribution and agree that ^177^Lu-PSMA-617 is unlikely to be cost-effective compared with cabazitaxel in the U.S. health care setting. However, the analysis likely underestimates the acquisition cost of ^177^Lu-PSMA-617 and may therefore understate how unfavorable the cost-effectiveness results are for ^177^Lu-PSMA-617 relative to cabazitaxel. We hope these considerations inform future economic evaluations of radiopharmaceuticals.

## DISCLOSURE

Kyle Flannery, Paul Cavanaugh, and Kevin Steele are employees of Sanofi, the manufacturer of Jevtana (cabazitaxel) injection, and may hold shares or options in the company. No other potential conflict of interest relevant to this article was reported.

## Notes

**Kyle Flannery**

*Sanofi, Morristown, New Jersey*

*E-mail: kyle.flannery@sanofi.com*

**Paul F. Cavanaugh**

*Sanofi, Morristown, New Jersey*

**Kevin Steele**

*Sanofi, Morristown, New Jersey*
